# Babao Dan attenuates acute ethanol-induced liver injury via Nrf2 activation and autophagy

**DOI:** 10.1186/s13578-019-0343-6

**Published:** 2019-10-01

**Authors:** Yang Yu, Zhi-qiang Tian, Lei Liang, Xue Yang, Dan-dan Sheng, Jian-xing Zeng, Xiao-yong Li, Rong-yu Shi, Zhi-peng Han, Li-xin Wei

**Affiliations:** 1 Tumor Immunology and Gene Therapy Center, Shanghai Eastern Hepatobiliary Surgery Hospital, The Second Military Medical University, 225 Changhai Road, Shanghai, 200438 China; 2 Department of Urology, Shanghai Tenth People’s Hospital, Tongji University School of Medicine, Shanghai, 200072 China

**Keywords:** Ethanol, Liver injury, Babao Dan, Oxidative stress, Autophagy

## Abstract

**Background:**

Babao Dan (BBD), a traditional Chinese medicine, has been used as a complementary and alternative medicine to treat multifarious liver diseases. In this study, we aimed to observe its protective effect on ethanol-induced liver injury and explore potential mechanisms.

**Methods:**

Mice pretreated with BBD (0.125, 0.25 and 0.5 g/kg BW) were administrated by ethanol gavage (5 g/kg BW). Liver injury biomarkers and hepatic redox parameters were evaluated by histopathology as well as serum and hepatic content analysis. AML-12 cell was also utilized to determine the efficacy of BBD against ethanol-induced hepatotoxicity.

**Results:**

Drunkenness experiment showed that the latency was significantly increased and the drunken sleep time was decreased in mice pretreated with BBD. We then found that BBD could reduce hepatic lipid peroxidation and steatosis induced by ethanol exposure. BBD could also suppress ethanol-induced depletion of hepatic antioxidant enzyme. Besides that, BBD treatment lessened the induction of hepatic cytochrome P450 2E1, a major contributor to ethanol-mediated oxidative stress, and up-regulated the expression of nuclear factor erythroid 2-related factor 2 and its two transcriptional targets hemeoxygenase-1 and glutamate-cysteine ligase catalytic subunit. Furthermore, autophagy induced by BBD contributed to hepatoprotection activity.

**Conclusions:**

Our results suggest that BBD can markedly dispel acute ethanol-induced hepatotoxicity through multiple pathways including attenuation of ethanol-mediated oxidative stress, enhancement of the oxidative defense systems and activation of autophagy.

## Introduction

Alcohol, as a well-known hepatotoxin, is widely consumed as a popular drink in the world [1]. There is a consistent epidemiological and clinical evidence that ethanol performs as a risk factor in various disorders, especially alcoholic liver disease (ALD) [2, 3]. As a worldwide health problem, ALD including alcoholic fatty liver, alcoholic steatohepatitis, alcoholic cirrhosis and increased risk of hepatocellular carcinoma in clinical spectrum [4] has been considered to be a major cause of mortality among people with alcohol abuse [5]. The toxicity of ethanol is mainly attributed to oxidative stress contributing to lipid peroxidation increase and hepatic oxidative defense systems decrease, which has been recognized to play pivotal roles in ALD initiation and progression [6, 7].

Ethanol ingestion induces the activation and expression of ethanol metabolizing enzyme cytochrome P450 2E1 (CYP2E1) which has been shown to play a major role in ethanol-induced oxidative stress and lipid accumulation [8–10]. The metabolism of ethanol via CYP2E1 leads to overproduction of reactive oxygen species (ROS), e.g. 1-hydroxyethyl radical (–OH), hydroxyl radical (H_2_O_2_) and superoxide radical (O_2_^−^) [11]. Importantly, ROS is considered as a major factor in a series of ethanol-induced pathologies [8, 12]. ROS can activate lipid peroxidation chain reactions, resulting in biomembrane dysfunction, membrane antioxidant enzymes inhibition, mitochondria damage and even cell death [12, 13]. The depletion of antioxidant enzymes including superoxide dismutase (SOD), glutathione peroxidase (GPx) and catalase (CAT) as well as the reduction of glutathione (GSH) level, leads to the oxidative stress in liver. Therefore, CYP2E1-generated oxidative stress inhibition as a potent strategy in preventing ethanol toxicity would likely be promising for ALD precaution.

In the past years, herbal medicines and compound preparations have received a considerable attention as potential agents for the prevention and treatment of ALD, ascribed to their multi-target actions and less side effects [14]. Babao Dan capsule (BBD), a mixed powder of traditional Chinese medicine, consists of eight constituents including natural Calculus Bovis, Snake Gall, Cornu Saigae Tataricae, Margarita, Moschus, Radix Notoginseng and so on. The formula of BBD was protected by Chinese Food and Drug Administration (CFDA). In clinic, BBD mainly used by oral medication for acute infectious viral hepatitis, acute cholecystitis and urinary tract infection caused by damp-heat brewing and binding. Our previous study has demonstrated that BBD can ameliorate liver injury and fibrosis in rat hepatic fibrosis model induced by diethylnitrosamine, and have no obvious side effect in normal rat livers [15]. However, the function and mechanism of BBD in preventing ethanol-induced liver injury are still unclear.

In this study, we demonstrated that BBD can ameliorate acute ethanol-induced hepatotoxicity through multiple pathways including attenuation of ethanol-mediated oxidative stress, enhancement of the oxidative defense systems and activation of autophagy. We also proved that BBD had no obvious side effect in normal mice livers. Upon our results, BBD may be a novel choice for protecting against acute ethanol-induced liver injury.

## Materials and methods

### Animals and treatments

Male C57BL/6 mice, 6–8 weeks old, weighting 20–22 g, were obtained from the Shanghai Experimental Animal Center of the Chinese Academy of Sciences, Shanghai, China. Experiments and procedures were approved by the Animal Ethics Committee of the Second Military Medical University. All animal handling and study procedures complied with the current Chinese regulation, GB14925-2010: Laboratory animal requirements of environment and housing facilities (Chinese version). For acute ethanol-induced liver injury model, ethanol binge was conducted as previously described [16] that mice were administrated by 50% (v/v) ethanol gavage at a dose of 5 g/kg BW after 6 h of fasting. BBD mixed powder, donated by Shanghai Pharmaceutical Group Inc., was dissolved in 0.9% saline at normal temperature and pressure to obtain BBD suspension with different concentrations for next experiments. To determine the preventive effect of BBD on acute ethanol liver damage, some mice were given BBD with increasing concentrations respectively by gavage prior to ethanol at 1 h apart. Control mice were given the same volume of normal 0.9% saline. They were sacrificed 16 h after ethanol administration. In the meanwhile, sleep latency (disappear of righting reflex) and sleep time on drunkenness (polysomnography) of mice was observed. Blood samples and liver tissues were collected at the end of experiments. The livers were dissected quickly, and portions of liver tissue were fixed in neutral buffered formalin, while others were frozen in liquid nitrogen. Plasma samples for further analysis were obtained by centrifugation (1750*g*, 15 min, 4 °C) and stored at − 80 °C.

### Cell culture and treatments

AML-12 cell line was obtained from the Chinese Academy of Sciences Cell Bank and cultured in DMEM supplemented with 10% fetal bovine serum and 1% penicillin streptomycin at 37 °C in a 5% CO_2_ humidified atmosphere. We collected portal vein serum from normal male C57BL/6 mice (normal-serum) and only BBD gavage treated male C57BL/6 mice (BBD-serum) at a dose of 0.5 g/kg BW for 1 h. Cells were treated with BBD-serum or other agents when they were 50–60% confluence. BBD-serum was added 1 h prior to the treatment of ethanol. Cells were harvested with lysis buffer after washing by PBS for three times, then were used for subsequent experiments.

### Reagents and biochemical assay

Chloroquine (CQ), 3-methyladenine (3-MA) were purchased from Sigma-Aldrich (St Louis, MO). The serum biochemical markers, including alanine aminotransferase (ALT), aspartate aminotransferase (AST) and triglyceride (TG) levels were measured with a biochemical autoanalyzer (Fuji Medical System, Tokyo, Japan). The activity of MDA, TG, ROS, GSH, GPx, SOD and CAT in liver tissues or cells were determined by kits (Nanjing Jiancheng Bioengineering Institute, Nanjing, China) according to the manufacturers’ instructions.

### Histopathology assessment

Liver tissues were sectioned and mounted on glass slides then stained with H&E. Each sample was observed at a 200× magnification of microscopic field in 10 randomly selected areas. The hepatic steatosis was evaluated by hepatic steatosis score, assigning from 0 to 3 where 0 = no steatosis, 1 = slight steatosis, 2 = moderate steatosis and 3 = severe steatosis. The liver cryostat section (8 mm) was stained with Oil Red O (Nanjing Jiancheng Bioengineering Institute, Nanjing, China) staining.

### RNA interference

The cells were transfected with Nrf2 siRNA (Santa Cruz Biotechnology, sc-37049) or non-targeting siRNA (Santa Cruz Biotechnology, sc-37007) using Lipofectamine 2000 according to the manufacturer’s instructions. Transfection efficiency in Cells were confirmed by western blot measurement.

### Transient transfection

Fugene HD transfection reagent (Calbiochem, La Jolla, CA) was used to transfect cells with GFP-LC3 expressing plasmids according to the manufacturer’s instructions. After initial treatment, autophagy was detected by counting the number of GFP-LC3-positive dots per cell under fluorescence microscope (Olympus IX71).

### Electron microscopic analysis

AML-12 cells were fixed in 2.5% glutaraldehyde in PBS (pH = 7.4) for 2 h at room temperature, then postfixed in 1% osmium tetroxide in water for 1 h, dehydrated in an ascending series of ethanol, and at last embedded in araldite (Basel, Switzerland). After solidified, 50 nm sections were cut on a LKB-I ultramicrotome and picked up on copper grids, post-stained with uranyl acetate and lead citrate, and observed in a Philips CM-120 TEM.

### Real time-PCR

Total RNA was isolated by using Trizol reagent (Invitrogen) according to the manufacturer’s specifications. cDNA was reverse-transcribed using the Revert Aid RT-PCR system (Fermentas, Pittsburgh, PA, USA). Real-time PCR was performed by mixing cDNA with primers and Maxima SYBR Green qPCR Master Mix (Applied Biosystems, Carlsbad, CA, USA). The sequences of the primers were as follows: CYP2E1 (forward: 5ʹ-CGTTGCCTTGCTTGTCTGGA-3ʹ; reverse: 5ʹ-AAGAAAGGAATTGGGAAAGGTCC-3ʹ), Nrf-2 (forward: 5ʹ-ACCAAGGGGCACCATATAAAA G-3ʹ; reverse: 5ʹ-CTTCGCCGAGTTGCACTCA-3ʹ), HO-1 (forward: 5ʹ-AGGTACAC ATCCAAGC CGAGA-30; reverse: 5ʹ-CATCACCAGCTTAAAGCCTTCT-3ʹ), GCLC (forward: 5ʹ-CTACCACG CAGTCAAGGACC-3ʹ; reverse: 5ʹ-CCTCCATTCAGTAACAACTGGAC-3ʹ) and β-actin (forward: 5ʹ-GGCTGTATTCCCCTCCATCG-3ʹ; reverse: 5ʹ-CCAGTTGGTAACAATGCCATGT-3ʹ). Real-time PCR was performed using a Stratagene Mx3000P Real-time PCR System with supplied software (Applied Biosystems), according to the manufacturer’s instructions. β-actin was used as internal control for RNA integrity and loading normalization.

### Western blot analysis

Cells and tissues were lysed in RIPA lysis buffer (Beyotime) with 1 mM PMSF. Equal amount of protein was separated by SDS-PAGE and transferred to NC membrane. The membranes were washed, blocked and incubated with specific primary anti-human antibodies against CYP2E1, p62/SQSTM1 (Cell Signaling Technology, Inc., Danvers, MA, USA), LC3 (Novus Biologicals, Littleton, CO), Nrf2, HO-1, GCLC and β-actin (Abcam, Cambridge, MA, USA), followed by incubation with horseradish peroxidase-conjugated secondary antibodies (Hangzhou HuaAn Biotech). Signals were visualized by chemiluminescent detection (Beyotime).

### Statistical analysis

All of the experiments were repeated at least three times. Final data were expressed as mean ± standard deviation (SD). Statistical analysis of the data was done by using GraphPad Prism 5. Student’s t-test was used to compare between mean values of two groups. Value of at least *P *< 0.05 was considered statistically significant.

## Results

### BBD protects against acute liver injury caused by ethanol exposure

**Fig. 1:**
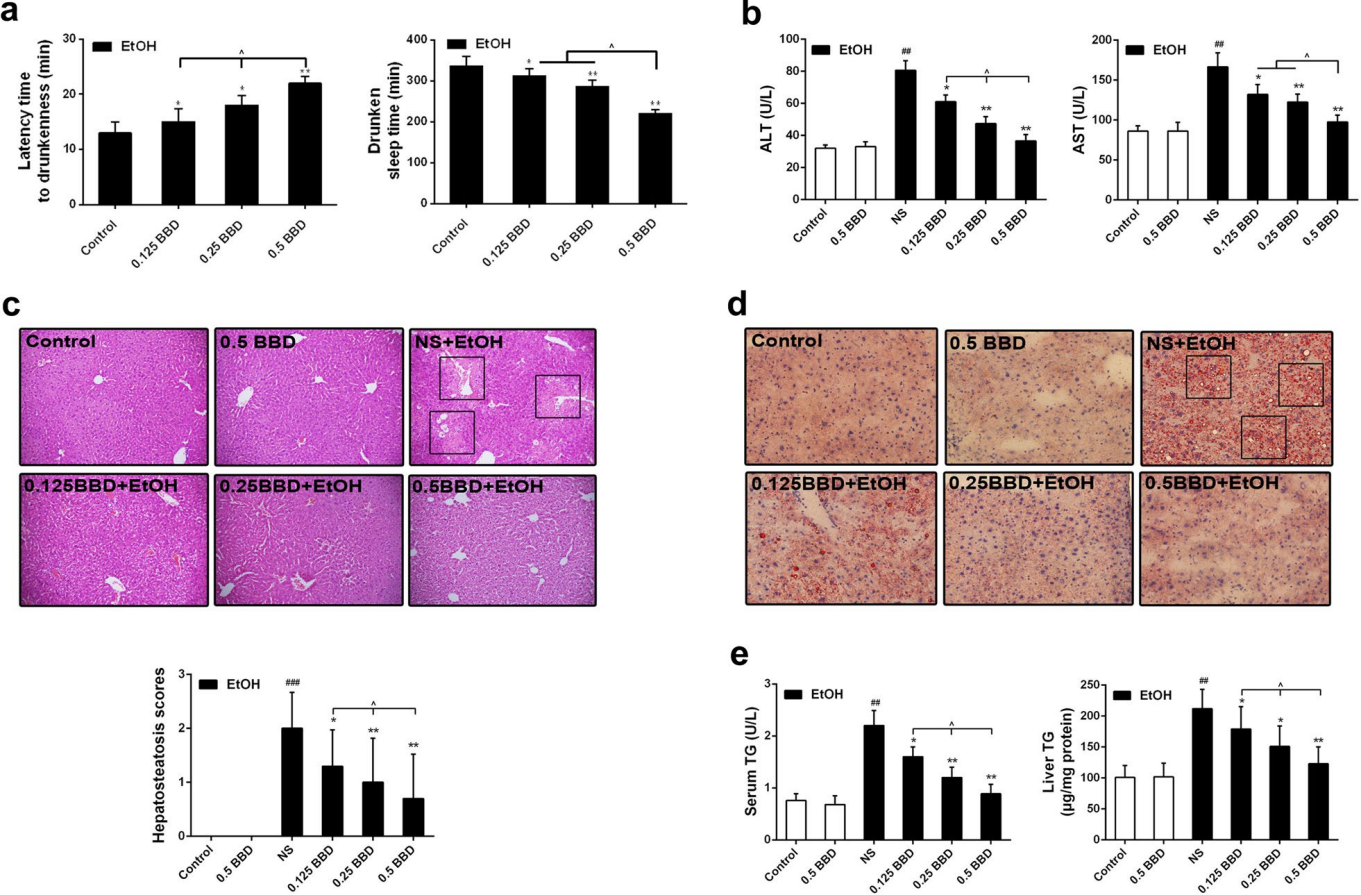
BBD protects against ethanol-induced liver injury. The mice were divided randomly into six groups (n = 10/group): control, normal 0.9% saline + ethanol, BBD (0.125 g/kg BW) + ethanol, BBD (0.25 g/kg BW) + ethanol, BBD (0.5 g/kg BW) + ethanol and BBD (0.5 g/kg BW). All mice survived the experimental period until sacrifice. **a** Both latency time to drunkenness (disappear of righting reflex) and drunken sleep time (polysomnography) were assessed. **b** Serum enzyme activities of aspartate ALT and AST were measured to reflect acute ethanol-induced liver injure. **c** Ethanol-induced histopathological changes in livers were presented by representative section photographs (original magnification: ×200) of H&E staining. The hepatic steatosis was also evaluated via hepatic steatosis scores ranging from 0, 1, 2 and 3 under a microscope. Mean rank was obtained for plotting. **d** Ethanol-induced lipid accumulation was demonstrated through representative Oil Red O-stained sections of livers (×200 magnification). **e** Liver and serum TG levels were determined as indicated methods. Values presented are from three replicates as mean standard ± deviation (SD), n = 10; ^##^*P* < 0.01, ^###^*P* < 0.001 compared to control; *P < 0.05, **P < 0.01 compared to NS group; ^^^*P* < 0.05. *BW* body weight, *NS* normal 0.9% saline, *EtOH* ethanol, *BBD* Babao Dan, *0.125 BBD* 0.125 mg/kg BBD, *0.25 BBD* 0.25 mg/kg BBD, *0.5 BBD* 0.5 mg/kg

To investigate the hepatoprotective effect of BBD, we first built an acute alcoholic liver damage model in mice and pretreated them with BBD to evaluate the efficacy in vivo. As shown in Fig. 1a, the latency to drunkenness was significantly increased and the drunken sleep time was reduced in mice pretreated with BBD when they suffered ethanol drunkenness experiment. These results indicate that BBD possesses an anti-drunk hangover effect potently. As the primary biochemical markers of liver injury, ALT and AST in serum was measured. Ethanol gavage caused a significant increase in serum ALT and AST, which could be prevented by BBD treatment, and BBD treatment alone did not change the level of them (Fig. 1b). Histopathological changes in liver are shown in Fig. 1c, acute ethanol exposure led to a potent hepatic steatosis, while BBD pretreatment obviously decreased ethanol induced hepatocyte steatosis. Noteworthily, BBD treatment alone had no detectable steatosis. Consistent with the pathogenic observations, BBD significantly reduced hepatic lipid accumulation induced by ethanol in liver (Fig. 1d). Furthermore, TG was measured in both blood and liver. As demonstrated in Fig. 1e, an effectively decreased TG level was detected in BBD pretreated mice when suffered ethanol gavage. These results suggest that BBD can significantly inhibit acute ethanol-induced hepatic function and tissue impairment.

### BBD prevents ethanol-induced hepatocyte oxidative stress damage in vivo

**Fig. 2:**
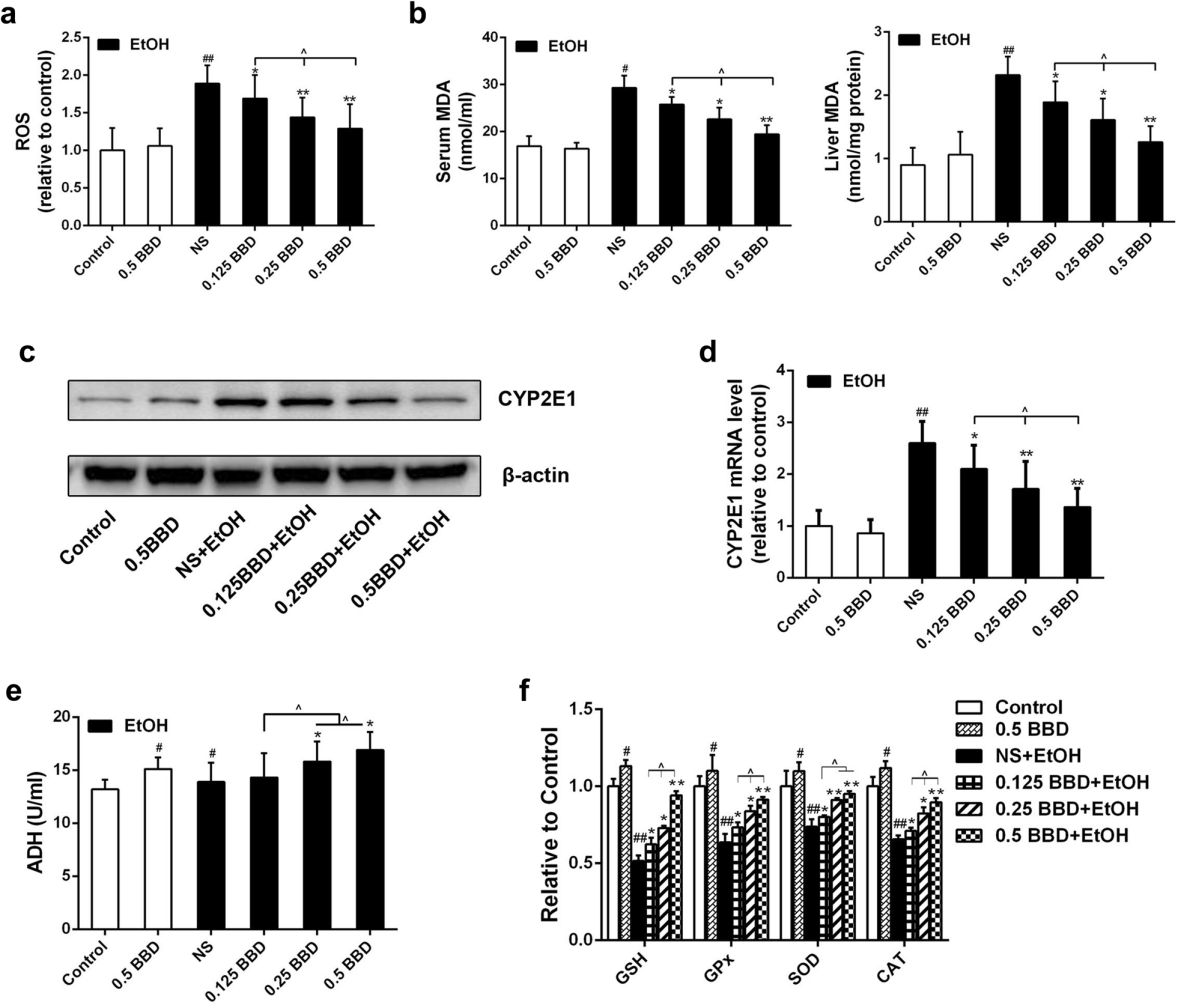
BBD prevents ethanol-induced hepatocyte oxidative damage. **a** ROS level in liver was measured by using 2ʹ,7ʹ-dichlorofluorescein diacetate (DCFH-DA) and analysed by the ELx 808 Universal Microplate Reader apply. **b** Liver and serum MDA levels were determined as indicated methods. Effects of BBD on CYP2E1 gene expression, including **c** immunoblot analysis and **d** real-time PCR analysis. Effects of BBD on liver ADH (**e**), GSH, GPx, SOD, CAT (**f**). The data presented are from three replicates as mean ± SD. ^#^*P* < 0.05, ^##^*P* < 0.01 compared to control; *P < 0.05, **P < 0.01 compared with NS group; ^^^*P* < 0.05. *NS* normal 0.9% saline, *EtOH* ethanol, *BBD* Babao Dan, *0.125 BBD* 0.125 mg/kg BBD, *0.25 BBD* 0.25 mg/kg BBD, *0.5 BBD* 0.5 mg/kg

As can be seen in Fig. 2a, ROS level in liver was significantly increased in ethanol group compared to control group, while BBD pretreatment group presented an obvious reduction. Also, ethanol exposure induced dramatic increase of malondialdehyde (MDA, an end-product of lipid peroxidation) in serum and liver tissue, which were strongly diminished by BBD treatment (Fig. 2b). CYP2E1 was reported as a major contributor to ROS generation and played a pivotal role in ethanol-induced fatty liver and oxidative stress [8–10]. As shown in Fig. 2c, d, ethanol administration greatly increased CYP2E1 expression compared to control and BBD pretreatment could effectively prevent the increase. Most ethanol-mediated effects depend on its metabolism. The first step of ethanol oxidization is mainly catalyzed by alcohol dehydrogenase (ADH). So we tested the ADH in liver and found that BBD could dramatically enhance ADH activation in certain dose (Fig. 2e). Effects of BBD on hepatic antioxidant enzymes (e.g. GPx, SOD and CAT) and non-enzymatic antioxidant (e.g. GSH) activities were also investigated. As shown in Fig. 2f, activities of GSH, GPx, CAT and SOD in ethanol exposure group were potently less than those in control group. However, these activities were effectively promoted by BBD. These findings indicate that BBD can alleviate hepatocyte lipid peroxidation and promote antioxidant capacity to resist ethanol exposure.

### BBD-serum inhibits liver cells oxidative stress induced by ethanol in vitro

**Fig. 3:**
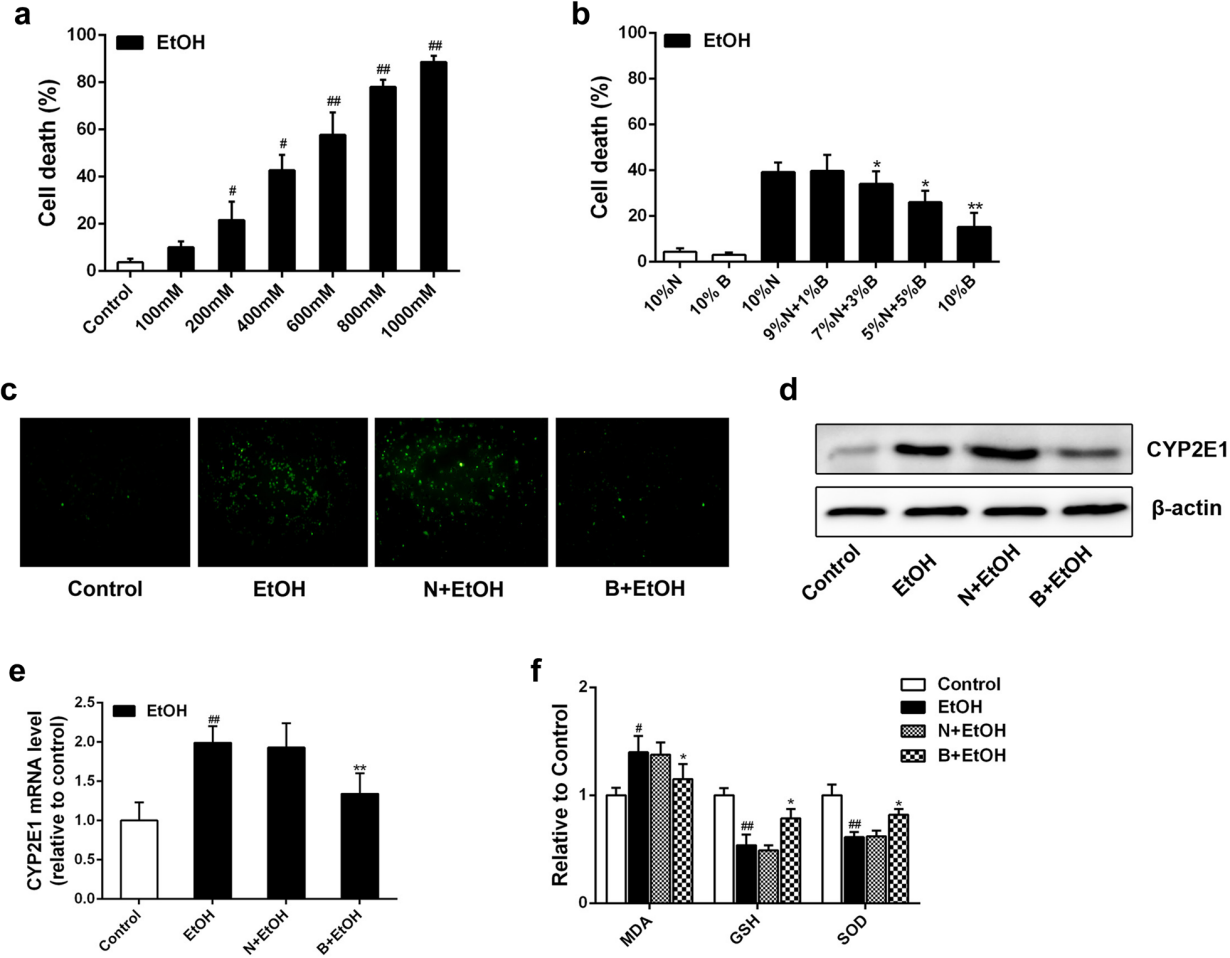
BBD-serum reduces ethanol-mediated AML-12 cell damage. Cell death measurement was performed with Annexin V staining. **a** AML-12 cells were treated with different concentrations (100 nM–1000 mM) of ethanol for 24 h. **b** AML-12 cells were pretreated with different concentrations of normal serum/BBD-serum for 1 h followed by administering with ethanol (400 mM) for 24 h. **c** DCFH-DA staining was utilized to detect intracellular ROS generation. The cells pretreated with 10% BBD-serum for 1 h was subsequently added 400 mM ethanol and then were incubated with 10 μM DCFH-DA for an additional 45 min at 37 °C. Fluorescence microscope analysis were performed to identify cellular ROS activity. **d**, **e** Effects of 10% BBD-serum on CYP2E1 expression in transcriptional and protein level when cells suffered 400 mM ethanol. **f** Influence of BBD-serum on MDA, GSH and SOD changes were presented. Cells were incubated with 400 mM ethanol in presence of 10% BBD-serum. Data present are from three replicates as mean ± SD. ^#^*P* < 0.05, ^##^*P* < 0.01, ^###^*P* < 0.001 compared to control; *P < 0.05, **P < 0.01 compared to 10%N + EtOH group. *N* normal-serum, *B* BBD-serum, *EtOH* ethanol

BBD-serum was added at the concentration of 1%, 3%, 5% and 10%, the rest of serum supplied by normal-serum to keep the serum concentration at 10% (v/v). Cell death induced by ethanol administration was then measured as described in previous studies [17]. Results showed that ethanol dose-dependently increased AML-12 cell death (Fig. 3a), while BBD-serum could effectively inhibit ethanol-induced cell death in dose-dependent manner (Fig. 3b). Following the results, we then performed the concentration of BBD-serum at 10% for the further study. As depicted in Fig. 3c, ethanol-induced overproduction of ROS was significantly suppressed when 10% BBD-serum was added before. Also, ethanol exposure induced a significant increase of CYP2E1 in both protein and mRNA level, which were strongly inhibited by BBD-serum (Fig. 3d, e). In addition, MDA and antioxidant markers (GSH and SOD) had the consistent consequence. These data suggest that BBD-serum leads to a significant reduction of ethanol-induced oxidative stress and increases the activity of antioxidant defenses.

### BBD ameliorates ethanol-induced cytotoxicity via Nrf2 activation

**Fig. 4:**
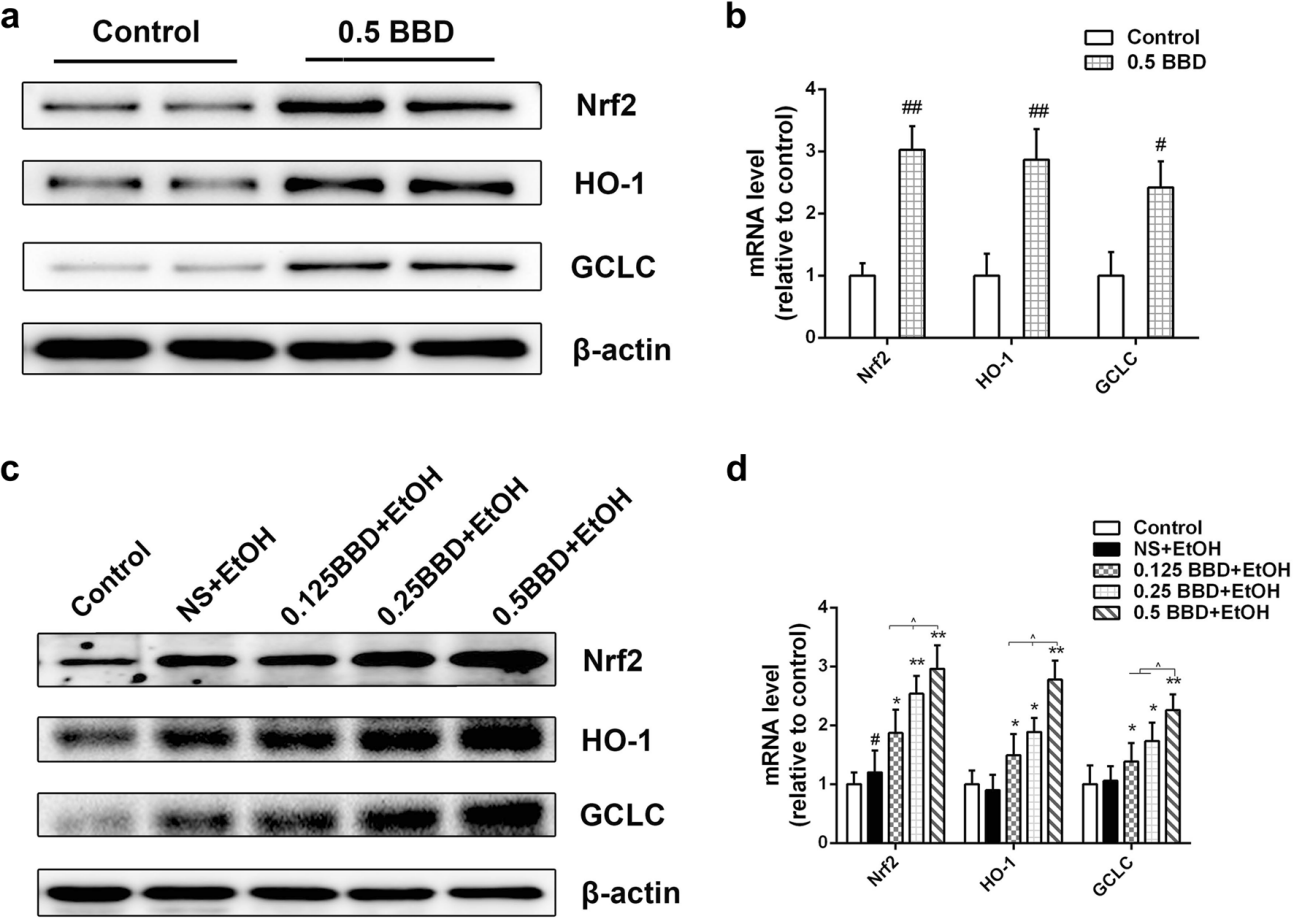
Nrf2 activation contributes BBD-mediated hepatoprotective effect. **a** Western blotting analysis of Nrf2 and the two transcriptional targets of HO-1 and GCLC expression in response to BBD (0.5 g/kg BW) alone. **b** Real-time PCR analysis of Nrf2 and the two downstream targets of HO-1 and GCLC transcription reacted to BBD treatment alone. Effects of BBD on related genes expression in **c** protein and **d** transcriptional level in liver. Experiments were repeated three times. Data were expressed as mean ± SD; ^#^*P* < 0.05, ^##^*P* < 0.01 compared to control; *P < 0.05, **P < 0.01 compared to NS + EtOH group; ^^^*P* < 0.05. *EtOH* ethanol, *0.125 BBD* 0.125 mg/kg BBD, *0.25 BBD* 0.25 mg/kg BBD, *0.5 BBD* 0.5 mg/kg

**Fig. 5:**
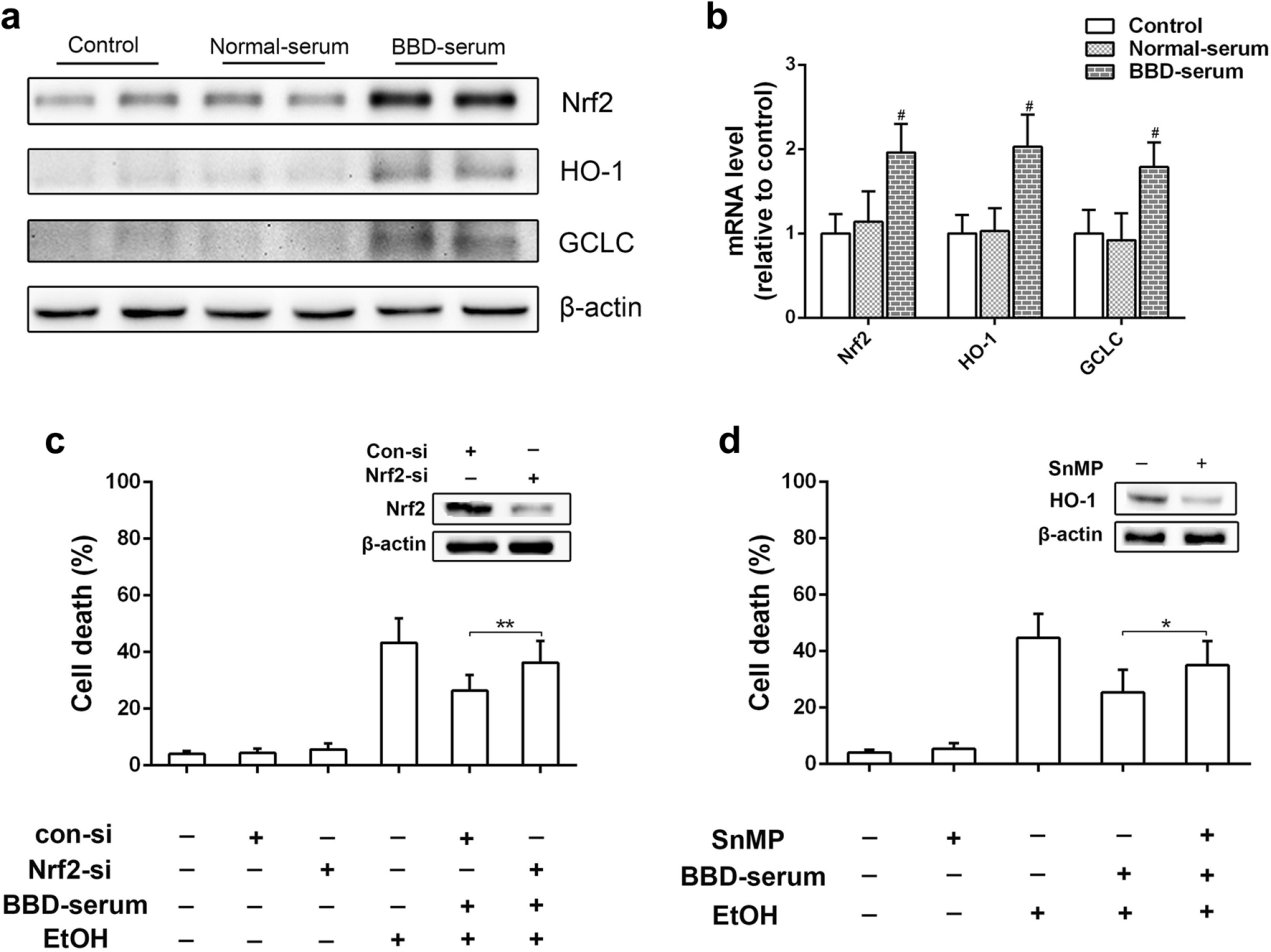
BBD-serum activates Nrf2 in cell culture model. BBD-serum up-regulates Nrf2 and its transcriptional targets HO-1 and GCLC in AML-12 cells. The cells were treated with 10% normal serum or 10% BBD-serum for 24 h and expression of Nrf2, HO-1 and GCLC were measured by **a** western blotting and **b** Real-time PCR. **c** Influences of Nrf2 knockdown on the protective effect of BBD-serum against ethanol-induced cell death. **d** Influences of HO-1 inhibition by its inhibitor SnMP on the protective effect of BBD-serum against ethanol-induced cell death. Values presented are from three replicates as mean ± SD. ^#^*P* < 0.05 compared to control; *P < 0.05, **P < 0.01. *EtOH* 400 mM ethanol

Nuclear factor erythroid 2-related factor 2 (Nrf2) is required for many antioxidant and detoxifying enzymes expression [18]. Recently, Nrf2 has also been reported as a new target to treat ALD [19]. Thus, the expression of Nrf2 and its downstream antioxidant protein hemeoxygenase-1 (HO-1) and glutamate-cysteine ligase catalytic subunit (GCLC) was examined. As shown in Fig. 4a, b, Nrf2, HO-1 and GCLC levels were apparently up-regulated in BBD treated mice compared to control. We also found that BBD pretreated mice showed an obvious increase of Nrf2, HO-1 and GCLC expression than those with ethanol treatment alone (Fig. 4c, d). To further determine the effect of BBD-serum on Nrf2 expression and its two downstream targets, in vitro studies were performed. We found that BBD-serum treated AML-12 cells presented increased expression of Nrf2, HO-1 and GCLC in protein and transcriptional levels (Fig. 5a, b). We also found the protective effect of BBD-serum against ethanol-induced cytotoxicity was dramatically attenuated when RNA interference was utilized to silence Nrf2 (Fig. 5c). Besides that, we obtained similar results when cells were treated with HO-1 inhibitor stannic mesoporphyrin (SnMP) (Fig. 5d). Together, our results suggest that the hepatoprotective effect of BBD is dependent on the regulation of Nrf2 pathway.

### BBD-serum activates autophagy in vitro

**Fig. 6:**
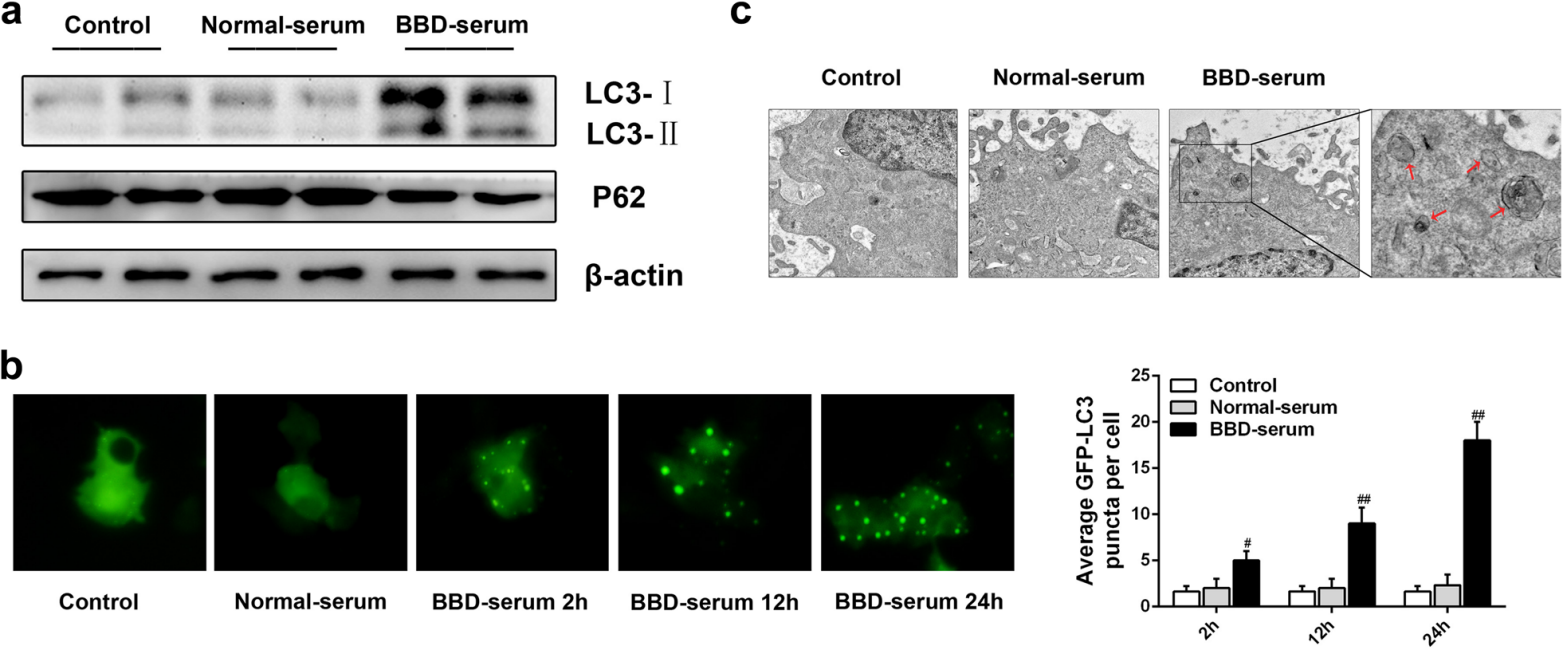
BBD-serum induces autophagy in AML-12 cells. **a** Immunoblots were used to analyze endogenous LC3 expression, and β-actin expression was used as control. Total protein extracts were analyzed by western blotting with antibody against p62. **b** GFP-tagged LC3 expression vector has been utilized to demonstrate the occurrence of autophagy and was detected using an inverted fluorescence microscope. **c** Representative electron micrographs (original magnification: ×3000) clearly exhibited the autophagic vacuoles in the cytoplasm content. Experiments were repeated three times. ^#^*P* < 0.05, ^##^*P* < 0.01 compared to control

Studies have reported that autophagy induction can reduce acute ethanol-induced hepatotoxicity and steatosis via removing damaged mitochondria and accumulated lipid droplets [20, 21]. We then explored if autophagy was involved in BBD-mediated hepatoprotection activity. As we know, the conjugated form of LC3 called LC3-II is targeted to the autophagosomal membrane and its accumulation is a symbol of autophagy activation [22]. Therefore, LC3-II used as a marker to monitor autophagic flux was analyzed by western blotting. As shown in Fig. 6a, the amount of LC3-II in BBD-serum treated AML-12 cells was significantly increased compared to normal-serum treated cells. We then examined the expression of P62, as another specific autophagy indicator [23], and obtained the consistent consequence. Additionally, Cells transfected with GFP-LC3 plasmid were analyzed by microscopy. BBD-serum obviously increased GFP-LC3 dots following up with time. Electron microscopic analysis was also employed to observe the autophagosome formation, suggesting the presence of characteristic double-membrane organelles in BBD-serum treated cells (Fig. 6c). All of these detections imply that BBD-serum can activate hepatocyte autophagy.

### Autophagy activation by BBD reduces ethanol-induced liver damage

**Fig. 7:**
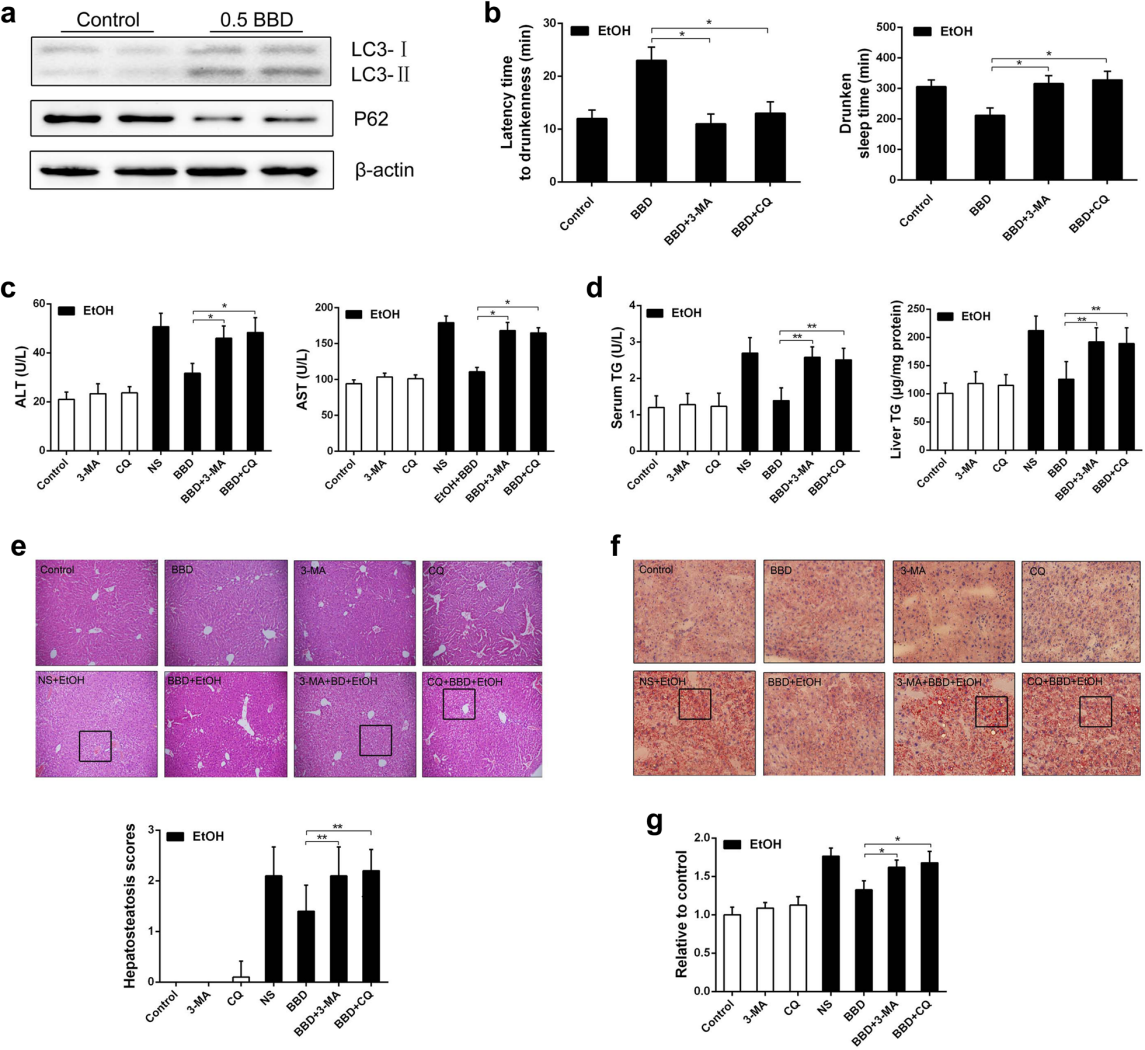
Autophagy activation reduces ethanol-induced liver damage. **a** Endogenous LC3 expression was detected by western blotting in liver with β-actin as a loading control. The conjugated and unconjugated form of LC3 is referred to as LC3-II and LC3-I, respectively. The protein level of p62 was also analyzed by western blotting. **b** C57BL/6 mice were pretreated with 3-MA (30 mg/kg) or CQ (50 mg/kg) intraperitoneally 30 min before the treatment of NS or BBD, and ethanol was administrated 1 h later as described in animals and treatments as well as the next steps. Both the latency time to drunkenness (disappear of righting reflex) and the drunken sleep time (polysomnography) were assessed. **c** Mice were treated as indicated and serum levels of ALT and AST were determined. **d** Influences of BBD-induced autophagy on serum and hepatic TG level. **e** One representative liver section performing H&E staining from each group is exhibited. Images are shown in ×200. **f** Effect of autophagy on liver lipid accumulation in mice administrated with ethanol was demonstrated through representative Oil Red O-stained liver sections (×200 magnification). **g** ROS level in liver. Results from three replicates were expressed as mean ± SD, n = 10; *P < 0.05, **P < 0.01; ^^^*P* < 0.05. *NS* normal 0.9% saline, *EtOH* ethanol, *BBD* 0.5 mg/kg Babao Dan

To further determine whether BBD prevents acute ethanol-induced liver injury by stimulating autophagy, we measured LC3-II level in BBD treated mice. Results showed that BBD markedly increased LC3-II expression and decreased P62 level (Fig. 7a). Moreover, the latency to drunkenness was obviously decreased and the drunken sleep time was increased when mice were pretreated with autophagy inhibitors 3-MA or CQ (Fig. 7b). Meanwhile, 3-MA or CQ pretreatment suppressed BBD mediated ALT and AST reduction (Fig. 7c). Similar results were obtained in serum or liver TG level when mice were pretreated with 3-MA or CQ (Fig. 7d). Even with BBD treatment, 3-MA or CQ pretreated mice showed a severe steatosis under ethanol administration (Fig. 7e). In addition, hepatic lipid accumulation and ROS production showed an obvious increase in 3-MA or CQ pretreated mice (Fig. 7f, g). Taken together, these results indicate that BBD can activate autophagy contributing to BBD-mediated hepatoprotection.

## Discussion

Growing evidence demonstrated that excessive ethanol consumption can lead to liver damage contributing to the pathogenesis of ALD [2–4]. However, there is still no universally accepted agents to prevent or reverse that worldwide, except abstinence from ethanol [24]. In current study, we firstly observed BBD pretreated mice showed the longer latency and the shorter drunken sleep time when they suffered ethanol drunkenness experiment (Fig. 1a). Then we found that BBD could effectively prevent mice with acute ethanol exposure against liver damage. As we know, ethanol-induced liver damage is associated with enhanced lipid peroxidation, formation of lipid radicals and a decrease in hepatic antioxidant protection [25]. Oxidative stress plays an critical role in pathogenesis of this process [5–7]. In our study, ethanol-induced hepatotoxicity was indicated by significant elevation of AST, ALT, and TG activities, hepatic lipid accumulation, as well as ROS and MDA production in vivo and in vitro. However, BBD would abolish them effectively. In addition, ethanol-induced cell death was also prevented by BBD-serum in a dose-dependent manner (Fig. 3b). All of our findings support that BBD could markedly reduce ethanol-induced hepatotoxicity.

CYP2E1 is considered as a key contributor to oxidative stress and potently increases ethanol-induced lipid accumulation in liver [8, 10]. It has been reported that a variety of enzymatic and nonenzymatic mechanisms are essential for endogenous antioxidant defense system to scavenge ROS and maintain cellular redox balance [2]. GSH has been implicated as a contributor to ROS detoxification and its shortage can be resulted from oxidative stress [26]. Apart from GSH, a great abundance of antioxidant enzymes including SOD, CAT and GPx can also weaken oxidative stress [27]. Thus, enhancing oxidative defense systems may prevent further liver injury during ethanol consumption. In present study, BBD significantly prevented ethanol-induced CYP2E1 up-regulation and reduction of GSH, GPx, CAT and SOD activities, indicating that BBD-mediated protective effect against alcoholic livery injury might be attributed to oxidative defense systems enhancement to resist oxidative stress.

In order to investigate whether the enhancement of oxidative defense systems was a direct response to BBD treatment or a secondary effect involved in oxidative stress, the expression of Nrf2 and its two downstream antioxidant protein HO-1 and GCLC was examined. Nrf2 is a key player of cellular antioxidant defense system, regulating the expression of many cytoprotective enzymes which results in cellular protection against a variety of insults produced by electrophilic and oxidative chemicals [18, 28]. Our data showed that BBD induced Nrf2 activation both in vivo (Fig. 4a) and in vitro (Fig. 5a). Moreover, the protective effect of BBD on ethanol-induced liver injury would be suppressed when Nrf2 silence was performed, clearly supporting involvement of Nrf2 pathway in BBD hepatoprotection (Fig. 5c, d). These results indicated that BBD could protect against ethanol-induced oxidative stress through directly acting on oxidative defense systems via Nrf2 pathway activation. In addition, we found that ethanol treatment alone also induced a slight increase in Nrf2 and its two transcriptional targets (Fig. 4c). This would be ascribed to mice self-protection performing stress reaction for a short time under acute ethanol exposure.

Autophagy is a critical cell survival mechanism in response to stress. Previous studies have reported that autophagy is a lysosomal degradation process that diminishes long-lived cellular proteins and removes damaged or excess organelles selectively [29–31]. Growing evidence indicates autophagy stimulation was an effective approach to alleviate liver injury, which demonstrates that autophagy can help to clear damaged mitochondria and lipid droplets to attenuate hepatocyte oxidative stress and steatosis [20, 21, 30, 32]. Here, we evaluated the effect of BBD on autophagy activation and showed that BBD can cause a significant induction of autophagy in vivo (Fig. 6a) and in vitro (Fig. 7a). Furthermore, lipid metabolism was reciprocally related to autophagy. Autophagy inhibition increased lipid accumulation while lipid accumulation repressed autophagy [20, 33, 34]. We then found that 3-MA or CQ significantly repressed BBD-induced hepatic steatosis depletion under ethanol exposure, and consistent consequence was obtained in histopathological changes and TG quantification. Moreover, the latency to drunkenness was obviously decreased and the drunken sleep time was increased when autophagy inhibition performed (Fig. 7b). That is consistent with previous study that autophagy could reduce acute ethanol-induced hepatotoxicity and steatosis [20]. It has been reported that autophagy inhibition induces ROS accumulation leading to cell damage [35, 36]. Consistent with these reports, our data demonstrated 3-MA or CQ exacerbated hepatic injury due to increased ROS level. Therefore, autophagy was proved as a survival mechanism for liver to protect against ethanol-induced hepatocyte injury. However, detail mechanisms about the reciprocal effect of ROS and autophagy need a further investigation.

In conclusion, major finding of present study is that BBD could effectively protect against acute ethanol-induced liver injury in mice through decreasing hepatocyte oxidative stress, increasing oxidative defense systems via Nrf2 pathway activation, as well as activating autophagy. As ALD are produced in a long developing and accumulating process, the effect of BBD on ethanol-induced chronic liver damage would be continued for a further study. All in all, our present results indicated that BBD may be a potential choice for the prevention of acute ethanol-induced liver injury.

## Data Availability

Please contact the corresponding author for data requests.

## Abbreviations

BBD: Babao Dan
ALD: alcoholic liver disease
CYP2E1: cytochrome P450 2E1
ROS: reactive oxygen species
SOD: superoxide dismutase
GPx: glutathione peroxidase
CAT: catalase
GSH: glutathione
CFDA: Chinese Food and Drug Administration
CQ: chloroquine
3-MA: 3-methyladenine
ALT: alanine aminotransferase
AST: aspartate aminotransferase
TG: triglyceride
MDA: malondialdehyde
H&E: hematoxylin and eosin
Nrf2: nuclear factor erythroid 2-related factor 2
GFP: green fluorescent protein
LC3: microtubule-associated protein 1 light chain 3
HO-1: hemeoxygenase-1
GCLC: glutamate-cysteine ligase catalytic subunit
SnMP: stannic mesoporphyrin
